# Hemodynamic and Functional Outcomes of Rate‐Adaptive and Physiologic Pacing in Heart Failure With Preserved Ejection Fraction: A Systematic Review and Meta‐Analysis

**DOI:** 10.1002/joa3.70504

**Published:** 2026-10-23

**Authors:** Gusti Ngurah Prana Jagannatha, David Yobel, Christo Timothy Mamangdean, Bagus Made Indrata Saputra, Anak Agung Ayu Dwi Adelia Yasmin, I Nyoman Wiryawan, I Made Putra Swi Antara

**Affiliations:** ^1^ Department of Cardiology and Vascular Medicine Udayana University/Prof. Dr. I.G.N.G. Ngoerah General Hospital Denpasar Bali Indonesia; ^2^ Clinical Clerkship Program, Faculty of Medicine Universitas Udayana, Prof. Dr. I.G.N.G. Ngoerah Central General Hospital Denpasar Bali Indonesia

**Keywords:** atrial pacing, chronotropic incompetence, heart failure with preserved ejection fraction, natriuretic peptides, quality of life, rate‐adaptive pacing

## Abstract

**Background:**

Exercise intolerance and poor quality of life remain challenges in HFpEF, driven by chronotropic incompetence and left atrial dysfunction. We evaluated whether rate‐adaptive/accelerated pacing (RAAP), targeting these mechanisms, improves hemodynamic, functional, and patient‐reported outcomes.

**Methods:**

PubMed, Embase, and the Cochrane Library were searched. Outcomes included NT‐proBNP, Minnesota Living with Heart Failure Questionnaire (MLHFQ) score, 6‐min walk distance (6MWD), E/e', and E/A. Within‐group pre–post changes and comparisons with usual care were synthesized separately. Effects are reported as mean differences (MD) or odds ratios (OR) with 95% confidence intervals (CI).

**Results:**

Eight reports involving 380 patients were included with myPACE overlap handled by one report per cohort per analysis. Within‐group, RAAP was associated with reductions in NT‐proBNP (MD −462.58 pg/mL; 95% CI −891.37 to −33.79), E/A (MD −1.01; −1.19 to −0.83), and E/e' (MD −3.18; −5.65 to −0.72), and improvements in 6MWD (MD +74.10 m; 36.34 to 111.86) and MLHFQ score (MD −17.40 points; −33.06 to −1.74). Compared with usual care, associations favored RAAP for NT‐proBNP (MD −108.51 pg/mL; −118.31 to −98.70), 6MWD (MD +73.36 m; 32.23 to 114.48), MLHFQ score (MD −16.52 points; −24.15 to −8.89), and AF burden (MD −6.13; −7.88 to −4.37). MACE also favored RAAP in an exploratory analysis (OR 0.47; 0.23 to 0.93), whereas peak VO_2_ did not differ significantly.

**Conclusions:**

RAAP was associated with favorable changes in hemodynamic and functional outcomes. These findings support further evaluation of individualized rate modulation in adequately powered randomized trials.

## Graphical abstract

In HFpEF, rate‐adaptive pacing (RAAP) delivers modest heart rate acceleration while preserving atrial‐ventricular synchrony, shortening diastasis and improving AV timing. This lowers left atrial filling pressure and pulmonary congestion, translating into reduced NT‐proBNP, E/e', and AF burden, alongside improved 6‐min walk distance and symptoms.

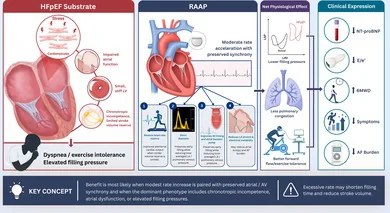

## 1 Introduction

Heart failure with preserved ejection fraction (HFpEF) accounts for approximately half of all heart failure cases and carries substantial morbidity, mortality, and healthcare burden, yet disease‐modifying options remain limited [1, 2]. Its pathophysiology is heterogeneous and includes impaired left ventricular (LV) relaxation and passive stiffening, elevated filling pressures, left atrial (LA) myopathy, pulmonary vascular remodeling, and blunted reserve during exertion [3, 4].

A frequently underappreciated contributor to exertional intolerance in HFpEF is chronotropic incompetence, that is, an inability to appropriately augment heart rate during exercise; in the small, stiff ventricle, stroke‐volume reserve is limited and cardiac output becomes increasingly rate‐dependent [5, 6]. This physiological insight provides the rationale for rate‐adaptive and moderately accelerated pacing (RAAP) as a mechanistic, device‐based intervention that seeks to restore heart‐rate reserve, shorten a disproportionately long diastolic interval, and, in selected phenotypes, reestablish atrial electromechanical synchrony [7, 8]. Unlike conventional pacing for bradyarrhythmia, RAAP is proposed to influence filling pressures and atrial mechanics even in patients without a standard pacing indication.

Early mechanistic and pilot studies support this rationale. Laurent et al. [9] reported that permanent LA pacing improved 6‐min walk distance and diastolic parameters in patients with atrial dyssynchrony, a proposed HFpEF substrate. Rate‐adaptive algorithm optimization improved exercise tolerance in paced HFpEF patients with permanent AF [10], and moderately accelerated personalized pacing improved quality of life, natriuretic peptides, and AF burden in the myPACE trial [11]. Conversely, the RAPID‐HF randomized trial found that rate‐adaptive atrial pacing did not improve exercise capacity and was accompanied by a reduction in stroke volume, underscoring that the net effect of heart‐rate modulation is phenotype‐dependent [12].

These divergent results, together with the diversity of pacing approaches (right atrial, Bachmann‐bundle/interatrial, and rate‐responsive or blood pressure‐adaptive algorithms), create genuine uncertainty about whether, and in whom, atrial pacing is beneficial in HFpEF. No prior quantitative synthesis has integrated the within‐group and controlled evidence across these strategies. The present systematic review and meta‐analysis therefore aimed to summarize the available evidence on the hemodynamic, functional, and patient‐reported effects of RAAP in HFpEF, while explicitly acknowledging the mechanistic heterogeneity of the interventions studied.

## 2 Methods

This systematic review and meta‐analysis was conducted and reported in accordance with the Preferred Reporting Items for Systematic Reviews and Meta‐Analyses (PRISMA) 2020 guidelines [13]. The review protocol was prospectively registered in PROSPERO (registration number CRD420261287328).

### 2.1 Search Strategy and Selection Criteria

PubMed (MEDLINE), Embase, and the Cochrane Central Register of Controlled Trials were systematically searched from inception to 30 October 2025. The search combined the following keywords and MeSH terms: (“Heart Failure with Preserved Ejection Fraction” OR “HFpEF”) AND (“Rate Adaptive Pacing” OR “Atrial Pacing” OR “Accelerated Pacing” OR “Physiologic Pacing”) AND (“NT‐proBNP” OR “Diastolic Function” OR “6‐min walk” OR “E/e' ratio” OR “E/A ratio” OR “Quality of Life” OR “Major Adverse Cardiac Event” OR “MACE”). Reference lists of relevant articles, prior reviews, and trial registries were hand‐searched to identify additional records. The identical final search date is reported in the Abstract and Methods to ensure reproducibility.

For the purposes of this review, RAAP was operationally defined as any rate‐adaptive or physiologic pacing strategy intended to augment or personalize heart rate and/or to restore physiologic atrial and atrioventricular activation in HFpEF. This umbrella definition encompasses rate‐responsive atrial pacing, moderately accelerated fixed‐rate atrial pacing, blood pressure‐adaptive atrial pacing, interatrial and conduction‐system pacing (Bachmann‐bundle, left atrial, or His‐bundle), and, in one study of patients with permanent atrial fibrillation, rate‐adaptive single‐chamber ventricular (VVIR) pacing. We deliberately adopted this broader category because the number of studies for any single technique is small; we recognize that these approaches target different pacing sites and are not mechanistically identical, and we address the implications of this grouping in the Discussion and Limitations.

Studies were eligible if they (i) enrolled adults (≥ 18 years) with HFpEF (LV ejection fraction ≥ 50%; or the study‐defined preserved‐EF threshold); (ii) applied an atrial‐based rate‐adaptive or accelerated pacing strategy as defined above; (iii) reported at least one outcome of interest (NT‐proBNP, MLHFQ, 6MWD, E/e', E/A, MACE, or AF burden); and (iv) used a randomized, prospective, or retrospective design. We excluded reports without extractable outcome data and non‐English publications. Given the small and emerging evidence base, and to avoid omitting pivotal randomized crossover data, we included peer‐reviewed randomized trials and prospective studies as well as prospectively conducted randomized crossover reports available in abstract form; this represents a deliberate refinement of the eligibility criteria relative to a conventional exclusion of all conference material, and studies available only as abstracts are explicitly identified and were interpreted with appropriate caution. Titles, abstracts, and full texts were screened independently by four reviewers, with disagreements resolved by discussion or by a fifth reviewer.

Handling of overlapping populations. Two included reports (Infeld 2023 and Infeld 2024) derive from the same myPACE randomized cohort and represent, respectively, the primary trial and an extended structural/long‐term analysis. To limit double‐counting, a given cohort contributed only once to any continuous within‐group or between‐group pooled estimate. For the MACE analysis, only one report per cohort was included (the primary myPACE trial and RAPID‐HF) to avoid double‐counting; because this outcome rests on only two independent cohorts, it is interpreted as exploratory. No other included studies were judged to report overlapping patient populations.

### 2.2 Data Extraction and Quality Assessment

Two reviewers independently extracted, using a standardized template: study design and year; sample size and demographics; pacing modality and configuration (e.g., right atrial, Bachmann‐bundle, His‐bundle, biventricular, or rate‐responsive algorithm); primary and secondary outcomes; and follow‐up duration. Where results were reported as medians with interquartile ranges or ranges, means and standard deviations (SDs) were estimated using the method of Wan et al. [14].

Risk of bias in randomized trials was assessed with the Cochrane Risk of Bias 2 (RoB 2) tool [15], and nonrandomized/observational studies with the Newcastle‐Ottawa Scale (NOS) [16]. For NOS, scores < 5 were considered high risk (low quality), 5–7 moderate, and > 7 low risk (high quality). For RoB 2, the overall judgment followed the recommended algorithm: a trial judged “low risk” in all domains was rated low risk overall, whereas “some concerns” in any single domain resulted in an overall rating of at least “some concerns”; the domain‐level rationale for each judgment is reported alongside the traffic‐light plots. Assessments were performed independently by four reviewers, with conflicts resolved by consensus.

### 2.3 Outcome Measurement and Analysis

Primary outcomes assessed within‐group change from baseline to the longest reported follow‐up in patients receiving RAAP: NT‐proBNP, 6MWD, MLHFQ, and the diastolic indices E/e' and E/A, all analyzed as continuous variables. For each study, preintervention and postintervention means and SDs were extracted; where multiple time points were available, the longest follow‐up was used. Because individual‐level change‐score SDs and the within‐patient baseline‐to‐follow‐up correlation were not available for most studies, we did not impute a correlation coefficient. Instead, baseline and follow‐up values were entered as separate groups (as reflected in the forest plots), which is conservative with respect to the variance of the paired difference and may widen confidence intervals; this assumption is acknowledged as a limitation.

Secondary outcomes evaluated between‐group differences (RAAP vs. usual care/standard therapy) in NT‐proBNP, MLHFQ, 6MWD, AF burden, MACE, and peak oxygen uptake (peak VO2)/VO2 at the anaerobic threshold as a measure of maximal exercise capacity. Continuous outcomes were expressed as mean differences and pooled by inverse‐variance weighting; MACE was pooled as an odds ratio using the Mantel–Haenszel method. For the peak‐capacity analysis, we note that the two contributing studies differed in design (a within‐patient crossover reporting VO2 at the anaerobic threshold and a parallel‐group comparison reporting the change in peak VO2); this analysis is therefore considered exploratory and interpreted with caution. To maintain analytical consistency across a small and clinically heterogeneous evidence base, a random‐effects (DerSimonian‐Laird) model was applied to all outcomes, irrespective of the observed I2; this harmonized approach matches the models displayed in all forest plots. Between‐study heterogeneity was quantified with the I2 statistic and Cochran *Q*. Sensitivity analyses (leave‐one‐out) were performed to assess the stability of pooled estimates. Formal small‐study/publication‐bias testing (Egger's test) was prespecified only for outcomes with ≥ 10 studies; because no outcome reached this threshold, funnel plots are presented for visual inspection only and are interpreted with caution. Prespecified subgroup analyses by pacing site, baseline rhythm, and baseline heart rate were not feasible owing to the small number of studies per outcome. Analyses were performed in Review Manager (RevMan) 5.4.

## 3 Results

### 3.1 Study Selection and Baseline Characteristics

The study‐selection process is summarized in the PRISMA flow diagram (Figure 1); records identified, duplicates removed, records screened, full texts assessed, and studies excluded with reasons are reported therein, and the counts are internally consistent (records screened = records assessed + excluded at screening; full texts assessed = included + full texts excluded). Reasons for full‐text exclusion included absence of extractable pre‐post or between‐group outcome data, populations not meeting the HFpEF definition, and interventions without a rate‐adaptive or accelerated pacing component. Eight reports met all eligibility criteria and were included in the qualitative and quantitative synthesis [9, 10, 11, 12, 17, 18, 19, 20].

**FIGURE 1:**
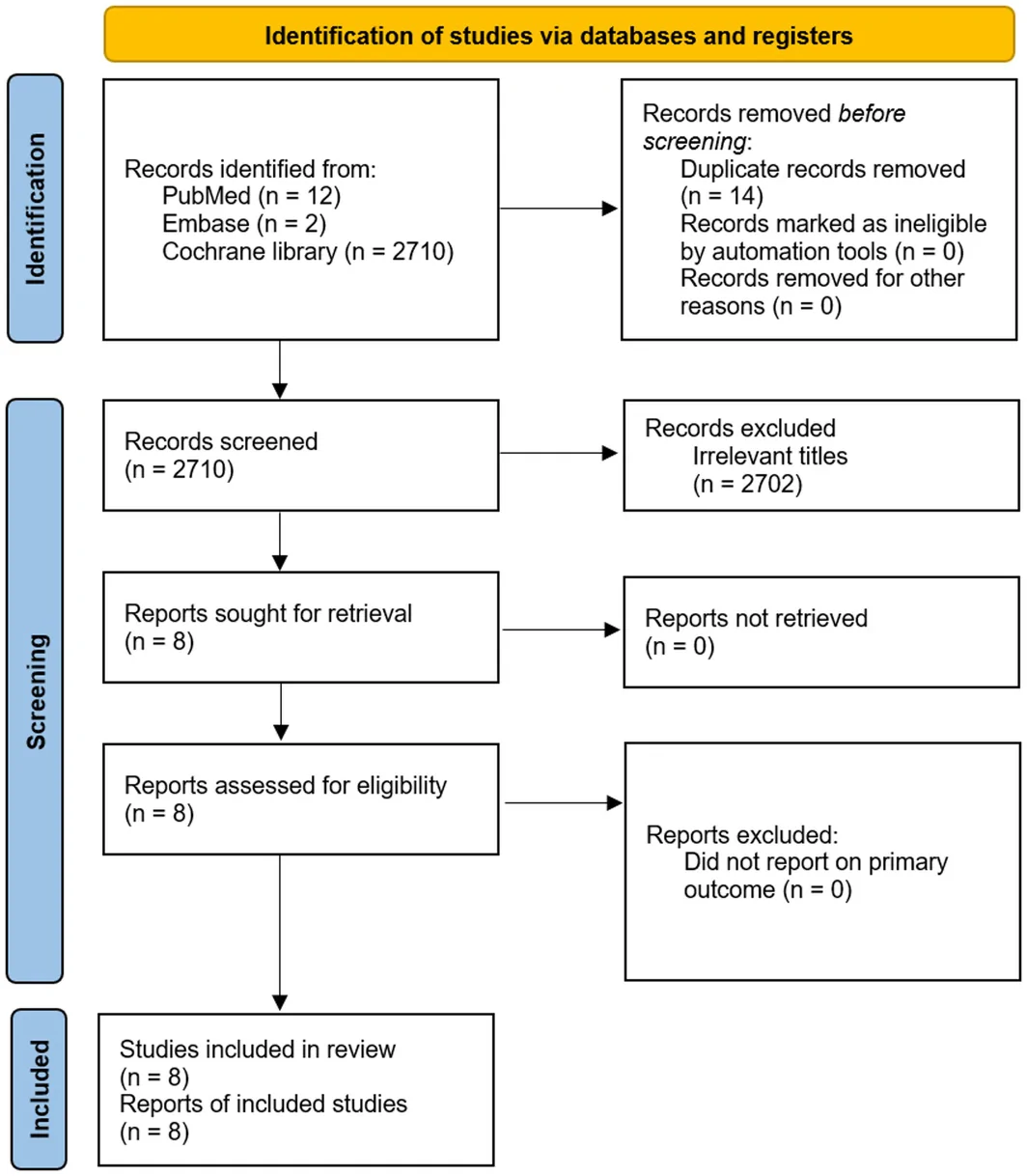
PRISMA 2020 flow diagram of study identification, screening, eligibility assessment, and inclusion, with reasons for full‐text exclusion.

Across the eight reports, 380 patients with HFpEF were studied (Table 1). As noted in the Methods, two reports (Infeld 2023 and Infeld 2024) originate from the same myPACE RCT; the count of unique participants is therefore lower than the summed enrolment, and this overlap was handled to avoid double‐counting within any single pooled outcome. Study size ranged from 6 [9] to 107 patients [17]. Mean age ranged from 46 years [18] to approximately 80 years [20]. Cohorts were frequently female‐predominant, with male representation ranging from 17% [9] to 83% [18]. Hypertension and AF were the most common comorbidities; AF was present in all participants in some cohorts [9, 10].

**TABLE 1:** Baseline characteristics of the included studies (total *N* = 380).

Study (year)	Country	Design	*N* (pace/control)	Mean age (*y*)	Male %	AF %	Pacing strategy	Follow‐up (mo)
Infeld 2023 (myPACE)	USA	RCT	100 (48/52)	75	55	NR	Personalized accelerated atrial pacing	36
Infeld 2024 (myPACE)^a^	USA	RCT	107 (50/57)	75	48.5	65.4	Atrial/conduction‐system/biventricular	12
Habel 2024 (PACE‐HFpEF)	USA	RCT crossover	20	78	NR	60	Conduction‐system (Bachmann/His)	NR
Laurent 2013	France	RCT	6	78	17	100	Dual‐chamber left atrial pacing	3
Reddy 2023 (RAPID‐HF)	USA	RCT crossover	29 (15/14)	66.5	47	24	Dual‐chamber rate‐adaptive	1
Serova 2020	Russia	RCT	54 (32/22)	69	47	100	VVIR rate‐adaptive	3
Wahlberg 2019	USA	Prospective	22	80	45	65	RA appendage/His‐Bachmann/RV	1.5
Subramanian 2025	India	RCT crossover	42 (21/21)	46	83	NR	Bachmann‐bundle area pacing	18.7

Abbreviations: NR, not reported; RA, right atrial; RV, right ventricular; VVIR, rate‐adaptive single‐chamber ventricular pacing.

^a^ Infeld 2024 reports an extended structural/long‐term analysis of the same myPACE cohort as Infeld 2023; the two are not independent samples, and a given cohort contributed only once to each pooled analysis.

Follow‐up ranged from about 1 month [12] to 36 months [11]. HFpEF was diagnosed using echocardiographic and clinical criteria broadly consistent with contemporary ESC/HFA definitions [8, 21]. Pacing strategies varied: right atrial or dual‐chamber rate‐responsive pacing was most common, while other studies used Bachmann‐bundle/interatrial pacing [18], blood pressure‐adaptive atrial pacing, or personalized accelerated pacing algorithms [10, 11].

### 3.2 Risk of Bias

The single nonrandomized/prospective cohort assessed with the NOS was rated low risk of bias, demonstrating adequate quality across the selection, comparability, and outcome domains (Figure 2A). Seven randomized trials were assessed with RoB 2 (Figure 2B): six were judged low risk across all domains, while Infeld 2023 raised “some concerns” in the randomization‐process domain (owing to limited reporting of allocation‐sequence concealment) and was therefore rated “some concerns” overall in accordance with the RoB 2 algorithm. Habel 2024 (PACE‐HFpEF) is available only in abstract form; its RoB 2 judgment is based solely on the methodological detail reported in that abstract, and, as with other abstract‐only data in this review, should be interpreted with corresponding caution given the limited reporting available for full appraisal.

**FIGURE 2:**
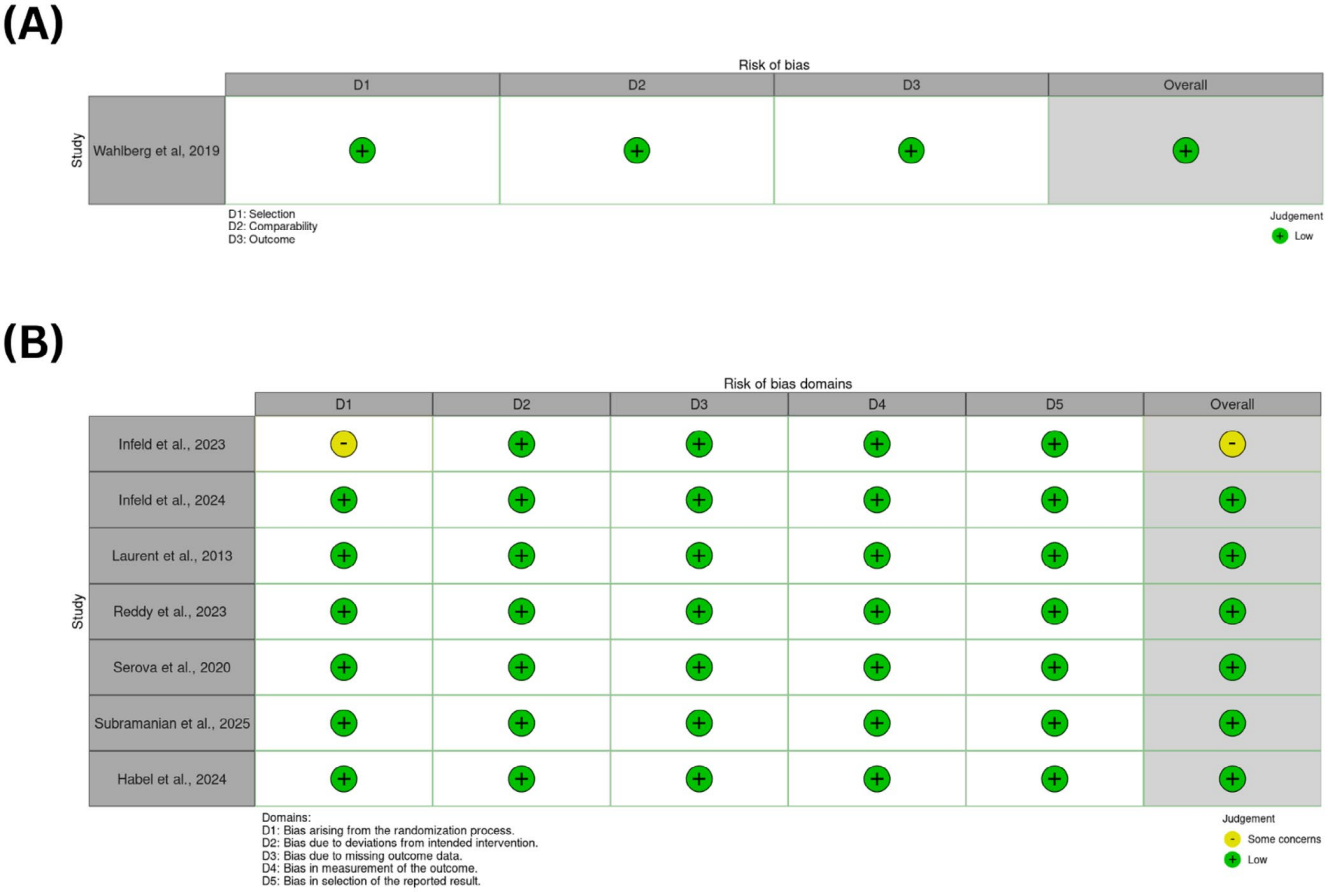
Risk‐of‐bias assessment (traffic‐light plots). (A) Nonrandomized/prospective cohort study assessed with the Newcastle‐Ottawa Scale. (B) Randomized trials assessed with the Cochrane Risk of Bias 2 (RoB 2) tool.

### 3.3 Primary (Within‐Group) Outcomes

Across six paced datasets from four studies (with the Wahlberg 2019 report contributing three pacing‐configuration subgroups) [9, 18, 19, 20], NT‐proBNP declined after pacing (MD −462.58 pg/mL; 95% CI −891.37 to −33.79; *p* = 0.034); between‐study heterogeneity was high (I2 = 94%) (Figure 3A).

**FIGURE 3:**
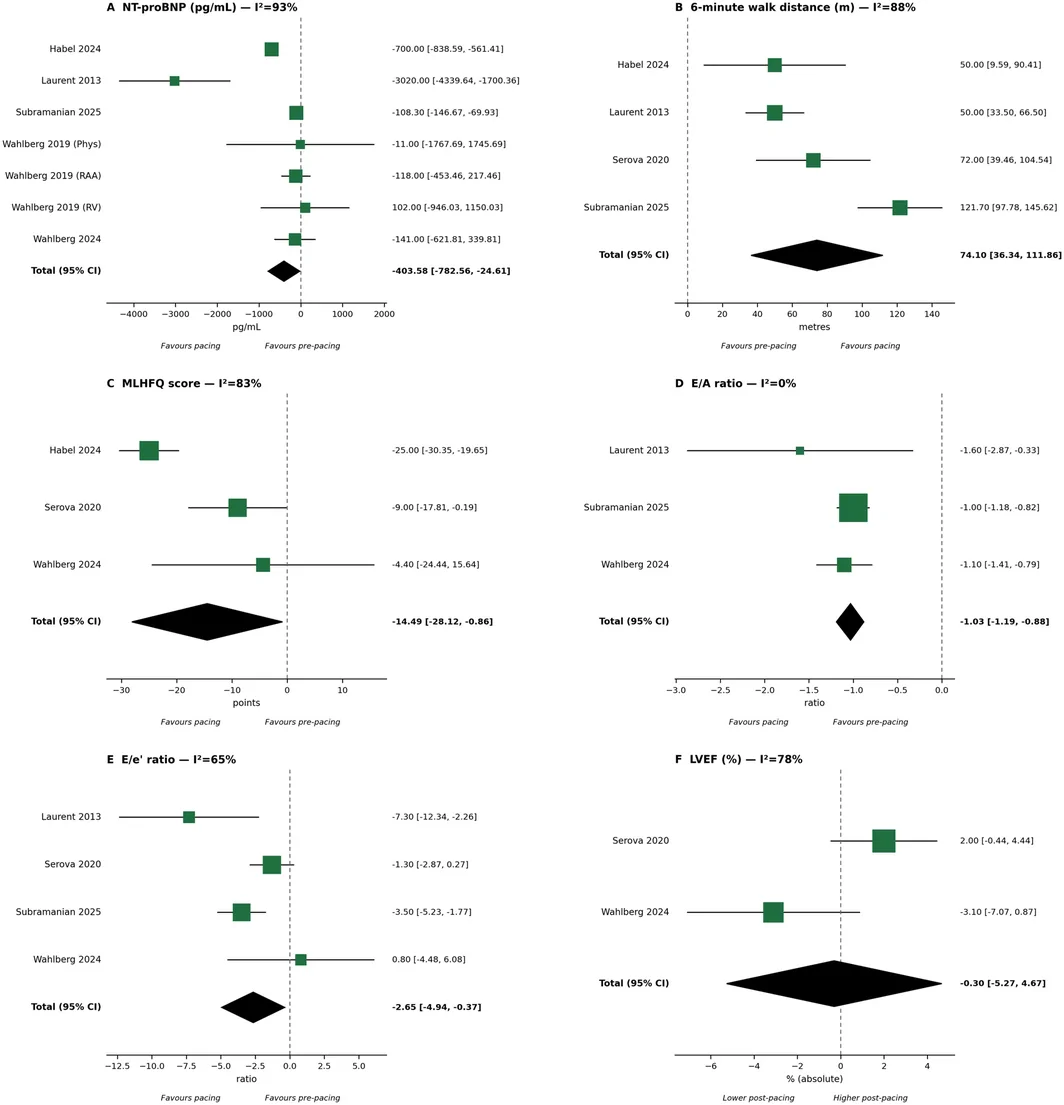
Within‐group (prepacing vs. postpacing) primary outcomes; random‐effects forest plots. (A) NT‐proBNP; (B) 6‐min walk distance (6MWD); (C) MLHFQ score; (D) E/A ratio; (E) E/e' ratio; (F) Left ventricular ejection fraction (LVEF).

Six‐minute walk distance increased after pacing across four studies [9, 10, 18, 19] (MD +74.10 m; 95% CI 36.34 to 111.86; *p* < 0.001), with consistent directionality despite considerable heterogeneity (I2 = 88%) (Figure 3B).

Health‐related quality of life, measured by the MLHFQ in two studies [10, 19], improved after pacing (MD −17.40 points; 95% CI −33.06 to −1.74; *p* = 0.029; I2 = 89%) (Figure 3C).

Two studies [9, 18] reported the E/A ratio, which fell after pacing (MD −1.01; 95% CI −1.19 to −0.83; *p* < 0.001) with no detectable heterogeneity (I2 = 0%) (Figure 3D).

The E/e' ratio, available in three studies [9, 10, 18], likewise decreased (MD −3.18; 95% CI −5.65 to −0.72; *p* = 0.011), with moderate heterogeneity (I2 = 71%) (Figure 3E).

### 3.4 Secondary (Between‐Group) Outcomes

In controlled comparisons, MACE was lower with pacing (OR 0.47; 95% CI 0.23–0.93; *p* = 0.03; I2 = 0%) [11, 12] (Figure 4A), based on two independent cohorts.

**FIGURE 4:**
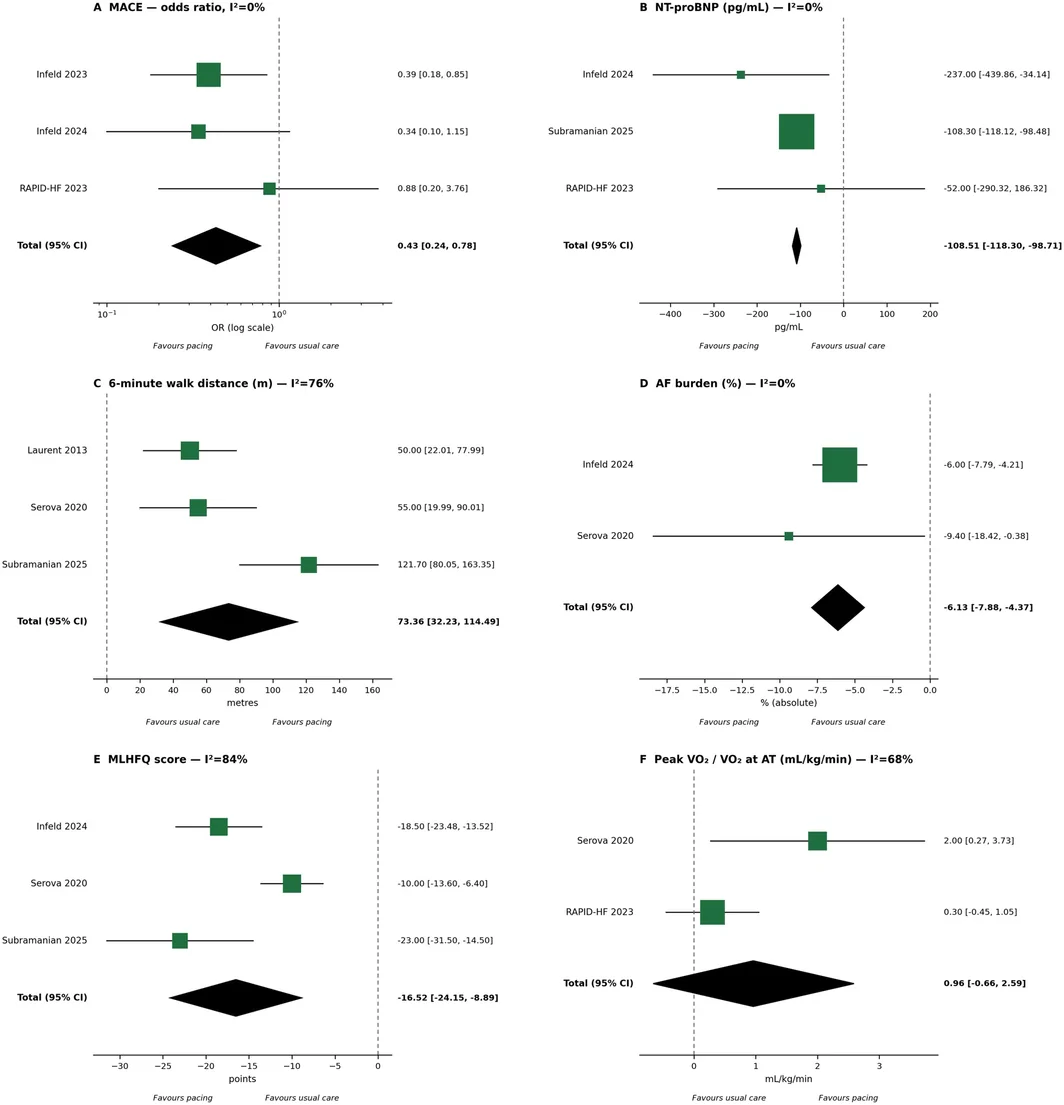
Between‐group outcomes, RAAP versus usual care; random‐effects forest plots. (A) major adverse cardiovascular events (MACE; odds ratio; primary analysis with one report per cohort); (B) NT‐proBNP; (C) 6MWD; (D) atrial fibrillation (AF) burden; (E) MLHFQ score; (F) peak VO2/VO2 at the anaerobic threshold (exploratory).

Three studies compared NT‐proBNP versus usual care [12, 17, 18]. The pooled MD was −108.51 pg/mL (95% CI −118.31 to −98.70; *Z* = 21.71; *p* < 0.00001; Tau2 = 0.00; Chi2 = 1.76, df = 2, *p* = 0.42; I2 = 0%) (Figure 4B). Subramanian 2025 contributed approximately 99.6% of the weight owing to its low reported variance; the pooled estimate is therefore effectively driven by a single study.

Three studies compared 6MWD versus usual care [9, 10, 18]. The pooled MD was +73.36 m (95% CI 32.23 to 114.48; *p* = 0.0005) across 56 RAAP and 47 control patients, with substantial heterogeneity (Tau2 = 1003.87; Chi2 = 8.50, df = 2, *p* = 0.01; I2 = 76%) (Figure 4C).

Two studies provided maximal‐capacity data versus usual care [10, 12]; the pooled analysis showed no significant difference in peak VO2/VO2 at the anaerobic threshold between RAAP and usual care (MD +0.96 mL/kg/min; 95% CI −0.66 to 2.59; *p* = 0.25; I2 = 68%) (Figure 4F).

Two studies reported between‐group AF burden [10, 17] (RAAP *n* = 60; control *n* = 67). The pooled MD was −6.13 (95% CI −7.88 to −4.37; *Z* = 6.85; *p* < 0.00001), with no detectable heterogeneity (Chi2 = 0.53, df = 1, *p* = 0.47; I2 = 0%) (Figure 4D). The estimate was dominated by the myPACE dataset (weight~96%).

Three studies compared MLHFQ versus usual care [10, 17, 18]. The pooled MD was −16.52 (95% CI −24.15 to −8.89; *Z* = 4.25; *p* < 0.0001), favoring RAAP, with considerable heterogeneity (Tau2 = 36.75; Chi2 = 12.18, df = 2, *p* = 0.002; I2 = 84%) (Figure 4E).

## 4 Discussion

This systematic review and meta‐analysis suggest that RAAP is associated with improved diastolic indices, functional capacity, and symptom burden in HFpEF, both in within‐group (pre‐post) analyses and in controlled comparisons with usual care. These associations are biologically coherent with the hypothesis that heart‐rate reserve, atrial mechanics, and diastolic loading are modifiable contributors to symptoms in HFpEF.

The reductions in NT‐proBNP, E/e', and E/A observed after pacing are directionally consistent with lower filling pressures and reduced LA wall stress, since E/e' correlates with invasively measured filling pressures and natriuretic peptides track wall stress [7, 22]. Because natriuretic peptides in HFpEF are relatively resistant to several pharmacological therapies, a pacing‐associated reduction is of mechanistic interest [3]. Nevertheless, the NT‐proBNP estimates were markedly heterogeneous (within‐group I2 = 94%) and for the between‐group comparison, effectively driven by a single study; both observations temper the strength of the inference.

Improvements in 6MWD (on the order of 70 m) and MLHFQ exceeded commonly cited thresholds for clinical relevance in heart failure, and paralleled the functional benefits reported for chronotropic and atrial‐timing optimization [5, 23]. Yet the substantial heterogeneity for these outcomes (I2 = 76%–89%) indicates that the magnitude of benefit is uncertain and likely phenotype‐dependent.

Beyond diastolic function, this synthesis did not permit a quantitative assessment of structural or systolic remodeling. Because left ventricular ejection fraction is preserved by diagnostic definition in HFpEF, it is neither the principal substrate nor an expected target of reverse remodeling, and only a single study reported paired ejection‐fraction data, precluding pooling. Adverse remodeling in HFpEF is instead dominated by diastolic stiffening and left atrial myopathy; the reductions in E/e', E/A, and NT‐proBNP observed here are therefore the more relevant indicators of favorable remodeling in this phenotype, reflecting lower filling pressures and reduced atrial wall stress. Dedicated structural and left atrial strain endpoints will be needed in future trials to characterize remodeling more directly.

### 4.1 Proposed Mechanisms Linking RAAP to Clinical and Functional Outcomes in HFpEF

Figure 5 illustrates the proposed mechanisms by which pacing may influence HFpEF physiology and translate into clinical and functional benefits. The framework begins with the HFpEF substrate: a small, stiff left ventricle with limited stroke‐volume reserve, an enlarged and stressed left atrium with impaired atrial function, and frequently coexisting chronotropic incompetence, a combination that culminates in elevated filling pressures, exertional dyspnoea, and exercise intolerance [3, 5, 7]. Against this substrate, RAAP delivers a moderate acceleration of atrial rate while preserving atrial and atrioventricular (AV) synchrony; it is through this physiologically constrained rate support, rather than aggressive rate elevation, that the four mechanistic pathways below are proposed to operate.

**FIGURE 5:**
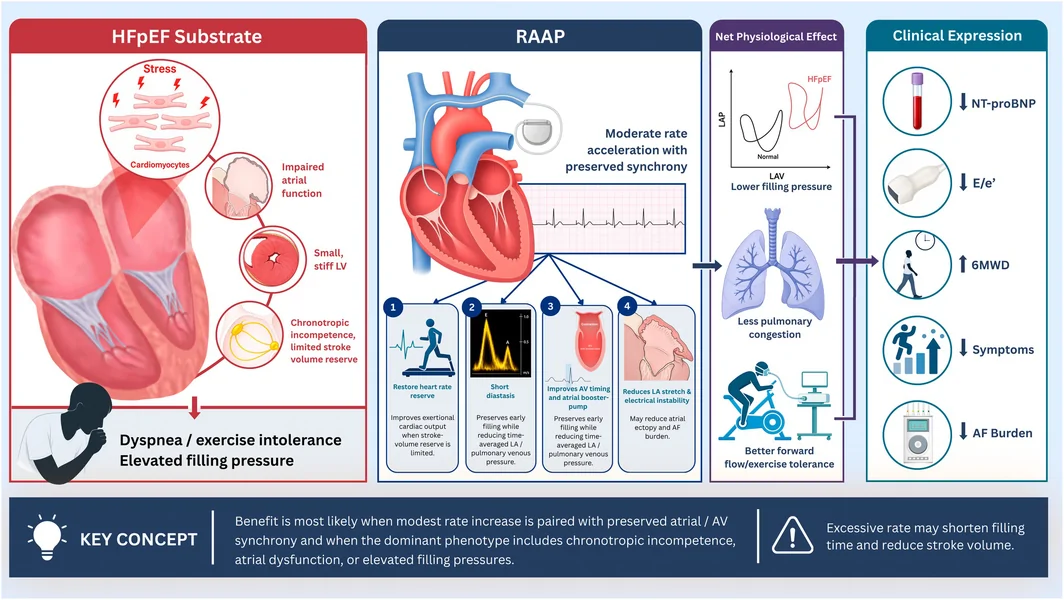
Central illustration. Proposed mechanisms linking rate‐adaptive/accelerated pacing (RAAP) to clinical and functional outcomes in HFpEF (see separate concept description).

First, RAAP restores heart‐rate reserve. Because the noncompliant HFpEF ventricle cannot substantially augment stroke volume during exertion, cardiac output becomes disproportionately rate‐dependent; when chronotropic reserve is impaired, the failure to raise heart rate appropriately limits exertional output and precipitates symptoms [5, 6]. By reestablishing an adequate rate response, RAAP can increase exertional cardiac output despite a fixed stroke volume, providing a physiological basis for the improvement in 6MWD and the reduction in symptom burden observed in this synthesis [10, 11].

Second, RAAP shortens diastasis while preserving early filling. At low resting rates, a stiff ventricle spends a prolonged interval in diastasis, a phase that adds little to filling yet exposes the left atrium and pulmonary veins to sustained elevated pressure. A modest rate increase preferentially truncates diastasis while sparing the early rapid‐filling phase, lowering time‐averaged LA and pulmonary venous pressure [3, 7]. This mechanism is supported by combined clinical‐computational data showing that accelerated atrial pacing reduces mean LA pressure [24], and it aligns closely with the reductions in E/e', E/A, and NT‐proBNP observed here, each of which tracks filling pressure and atrial wall stress [22].

Third, RAAP improves AV timing and the atrial booster‐pump contribution. In HFpEF, late diastolic (atrial) filling is disproportionately important because early filling is impaired; loss of optimal AV timing or interatrial conduction delay degrades the atrial contribution and further elevates LA pressure [7, 9]. Atrial‐based and interatrial (Bachmann‐bundle) pacing can re‐coordinate atrial activation and restore effective AV timing, enhancing late diastolic filling and lowering LA pressure, a plausible explanation for the diastolic and symptomatic gains seen with resynchronization‐oriented strategies [9, 18].

Fourth, RAAP may reduce LA stretch and electrical instability. Chronic pressure‐ and volume‐related atrial stretch promotes atrial myopathy and establishes the substrate for atrial fibrillation, which in turn accelerates HFpEF progression [7, 25]. By lowering atrial loading and, in some configurations, by increasing atrial pacing burden that suppresses ectopy, RAAP may attenuate atrial ectopy and AF burden, consistent with the reduction in AF burden found in the controlled comparisons; autonomic effects of a more physiological heart‐rate profile and a more favorable ventricular‐arterial coupling may contribute further in selected patients [10, 17, 26].

Taken together, these pathways are proposed to generate a coherent set of net physiological effects, namely lower filling pressure, less pulmonary congestion, and better forward flow and exercise tolerance, which map onto the clinical signals detected in this meta‐analysis: lower NT‐proBNP, lower E/e', greater 6MWD, reduced symptom burden (MLHFQ), and lower AF burden [10, 11, 18]. The concordance between this mechanistic scheme and the direction of the pooled estimates strengthens the biological plausibility of the observed associations, although it does not by itself establish causation.

These benefits are conditional rather than universal, and Figure 5 deliberately frames RAAP as a phenotype‐dependent therapy. Benefit is most likely when a modest rate increase is paired with preserved atrial and AV synchrony and when the dominant phenotype includes chronotropic incompetence, atrial dysfunction, or elevated filling pressures [5, 7]. Conversely, excessive rate acceleration shortens the filling period and can reduce stroke volume, offsetting any gain in output, as demonstrated by the neutral primary endpoint of the RAPID‐HF trial in patients selected primarily for chronotropic incompetence [12]. This physiology is mirrored in the present synthesis, in which peak VO2 was not significantly improved, indicating that the diastolic, biomarker, and symptomatic benefits of RAAP were not accompanied by a gain in maximal aerobic capacity and should not be extrapolated to it. The therapeutic effect of RAAP is therefore best understood as narrow and phenotype‐specific and should not be overgeneralized across the heterogeneous HFpEF population.

### 4.2 Comparison With Pharmacological Rate Control

These observations should be viewed alongside the ongoing re‐appraisal of heart‐rate‐lowering therapy in HFpEF. Beta‐blockers, prescribed in a large proportion of HFpEF patients largely by extrapolation from HFrEF, lack a clear mortality benefit in preserved‐EF populations and may, in some phenotypes, aggravate chronotropic incompetence [27, 28]. Emerging data and expert perspectives suggest that liberating rather than suppressing heart rate may benefit selected patients with the smallest, stiffest ventricles [29, 30, 31]. RAAP and judicious deprescription of negative chronotropes may thus be complementary expressions of the same principle: matching heart rate to the physiology of the individual ventricle rather than applying a uniform target [32].

### 4.3 Heterogeneity and Sources of Variability

Substantial heterogeneity was present for several outcomes (notably NT‐proBNP, 6MWD, and MLHFQ). Plausible contributors include differences in pacing site and mode (right atrial, interatrial/Bachmann‐bundle, conduction‐system/His‐bundle, rate‐responsive or blood pressure‐adaptive, and, in one study of permanent atrial fibrillation, rate‐adaptive single‐chamber ventricular [VVIR] pacing), patient phenotype (including the hypertrophic‐cardiomyopathy/HFpEF cohort), baseline rhythm and chronotropic status, baseline heart rate, and follow‐up duration. Because the number of studies per outcome was small, prespecified subgroup and meta‐regression analyses were not feasible; we therefore report heterogeneity transparently rather than attempting under‐powered subgroup inference. This mechanistic and methodological heterogeneity is also the reason we treat the umbrella “RAAP” category as a family of related but non‐identical interventions spanning atrial, conduction‐system, and ventricular rate‐adaptive pacing.

### 4.4 Limitations

This synthesis should be interpreted in light of several considerations that also help delineate where future work is most needed. As the first quantitative synthesis of an emerging device‐based strategy, the review is built on a small but growing evidence base, with individual outcomes informed by two to five studies; this reflects the novelty of the field rather than a deficiency of the synthesis, but it did preclude prespecified subgroup analyses, meta‐regression, and formal small‐study/publication‐bias testing (Egger's test requires ≥ 10 studies), and funnel plots are therefore presented descriptively only. The pooled interventions are mechanistically related but not identical: they span right atrial, interatrial (Bachmann‐bundle), conduction‐system (His‐bundle), and, in one study of permanent atrial fibrillation, rate‐adaptive single‐chamber ventricular (VVIR) pacing. Grouping these under a single RAAP label, although pragmatic and predefined, therefore reflects heart‐rate–modulating and physiologic pacing strategies collectively rather than a single atrial‐pacing modality, and may obscure strategy‐specific effects; we accordingly treated RAAP as a family of interventions and interpreted the more heterogeneous outcomes (notably NT‐proBNP) conservatively. Two reports derive from the same myPACE cohort; we constrained each cohort to contribute only once to every pooled analysis. The MACE analysis, in particular, rests on only two independent cohorts with few events and is therefore interpreted as exploratory. Some data were available only in abstract form and were interpreted with corresponding caution. For within‐group analyses, baseline and follow‐up values were entered as separate groups because change‐score standard deviations and the baseline‐to‐follow‐up correlation were unavailable, a deliberately conservative approach that tends to widen rather than narrow the confidence intervals. Finally, follow‐up was predominantly short to intermediate, so durability and long‐term safety remain to be established.

Notwithstanding these considerations, several features support the credibility of the findings. The review was conducted within the PRISMA 2020 framework, with independent screening, data extraction, and risk‐of‐bias assessment by multiple reviewers and with prespecified outcomes and models. Importantly, the direction of effect was consistent across independent cohorts and across different pacing strategies even where the magnitude varied, and leave‐one‐out sensitivity analyses preserved the pooled estimates. Rather than establishing efficacy, these convergent and internally consistent signals identify a biologically coherent and testable hypothesis and, in doing so, specify the phenotypes, pacing strategies, and endpoints that adequately powered randomized trials should now prioritize.

### 4.5 Clinical and Research Implications

Taken together, these findings support RAAP as a mechanistically plausible, hypothesis‐generating strategy in HFpEF rather than an established therapy. Future work should prioritize adequately powered randomized trials in phenotypically characterized populations, with pre‐specified stratification by chronotropic competence, atrial conduction and strain, and baseline rhythm, and with standardized pacing protocols and outcome definitions. Integration of ambulatory or implantable sensors may permit individualized, closed‐loop rate optimization. The central clinical question is shifting from how much to slow the heart to how to match heart rate to the physiology of the individual stiff ventricle [29, 31].

## 5 Conclusion

In this systematic review and meta‐analysis, RAAP was associated with improved echocardiographic diastolic indices (E/e', E/A), lower NT‐proBNP, greater 6‐min walk distance, and better quality of life in patients with HFpEF, together with lower AF burden and fewer MACE in controlled comparisons. These associations are consistent with a mechanistic effect on heart‐rate reserve, atrial mechanics, and diastolic loading. However, given the small number of studies, heterogeneous and nonidentical pacing strategies, partial cohort overlap, and predominantly short follow‐up, these findings should be regarded as hypothesis‐generating. These results require confirmation in adequately powered, phenotype‐enriched randomized trials before informing routine clinical practice.

## Funding

The authors have nothing to report.

## Ethics Statement

The authors have nothing to report.

## Consent

The authors have nothing to report.

## Conflicts of Interest

The authors declare no conflicts of interest.

## Supporting information


**Figures S1–S11:** Funnel plots for each pooled outcome, presented for visual inspection only (formal small‐study/publication‐bias testing was not performed because no outcome included ≥ 10 studies).

## Data Availability

The data that support the findings of this study are available from the corresponding author upon reasonable request.

## References

[1] T. E. Owan , D. O. Hodge , R. M. Herges , S. J. Jacobsen , V. L. Roger , and M. M. Redfield , “Trends in Prevalence and Outcome of Heart Failure With Preserved Ejection Fraction,” New England Journal of Medicine 355, no. 3 (2006): 251–259, 10.1056/NEJMoa052256. 16855265

[2] S. M. Dunlay , V. L. Roger , and M. M. Redfield , “Epidemiology of Heart Failure With Preserved Ejection Fraction,” Nature Reviews. Cardiology 14, no. 10 (2017): 591–602, 10.1038/nrcardio.2017.65. 28492288

[3] B. A. Borlaug , “Evaluation and Management of Heart Failure With Preserved Ejection Fraction,” Nature Reviews. Cardiology 17, no. 9 (2020): 559–573, 10.1038/s41569-020-0363-2. 32231333

[4] B. A. Borlaug and M. M. Redfield , “Diastolic and Systolic Heart Failure Are Distinct Phenotypes Within the Heart Failure Spectrum,” Circulation 123, no. 18 (2011): 2006–2013, 10.1161/CIRCULATIONAHA.110.954388. PMC3420141 21555723

[5] P. H. Brubaker and D. W. Kitzman , “Chronotropic Incompetence: Causes, Consequences, and Management,” Circulation 123, no. 9 (2011): 1010–1020, 10.1161/CIRCULATIONAHA.110.940577. PMC3065291 21382903

[6] B. A. Borlaug , V. Melenovsky , S. D. Russell , et al., “Impaired Chronotropic and Vasodilator Reserves Limit Exercise Capacity in Patients With Heart Failure and a Preserved Ejection Fraction,” Circulation 114, no. 20 (2006): 2138–2147, 10.1161/CIRCULATIONAHA.106.632745. 17088459

[7] Y. N. V. Reddy , M. Obokata , F. H. Verbrugge , G. Lin , and B. A. Borlaug , “Atrial Dysfunction in Patients With Heart Failure With Preserved Ejection Fraction and Atrial Fibrillation,” Journal of the American College of Cardiology 76, no. 9 (2020): 1051–1064, 10.1016/j.jacc.2020.07.009. PMC7455760 32854840

[8] B. Pieske , C. Tschope , R. A. de Boer , et al., “How to Diagnose Heart Failure With Preserved Ejection Fraction: The HFA‐PEFF Diagnostic Algorithm of the Heart Failure Association (HFA) of the European Society of Cardiology (ESC),” European Heart Journal 40, no. 40 (2019): 3297–3317, 10.1093/eurheartj/ehz641. 31504452

[9] G. Laurent , J. C. Eicher , A. Mathe , et al., “Permanent Left Atrial Pacing Therapy May Improve Symptoms in Heart Failure Patients With Preserved Ejection Fraction and Atrial Dyssynchrony: A Pilot Study Prior to a National Clinical Research Programme,” European Journal of Heart Failure 15, no. 1 (2013): 85–93, 10.1093/eurjhf/hfs150. 23018991

[10] M. Serova , D. Andreev , I. Giverts , et al., “A New Algorithm for Optimization of Rate‐Adaptive Pacing Improves Exercise Tolerance in Patients With HFpEF,” Pacing and Clinical Electrophysiology 43, no. 2 (2020): 223–233, 10.1111/pace.13857. 31876292

[11] M. Infeld , K. Wahlberg , J. Cicero , et al., “Effect of Personalized Accelerated Pacing on Quality of Life, Physical Activity, and Atrial Fibrillation in Patients With Preclinical and Overt Heart Failure With Preserved Ejection Fraction: The myPACE Randomized Clinical Trial,” JAMA Cardiology 8, no. 3 (2023): 213–221, 10.1001/jamacardio.2022.5320. PMC9996402 36723919

[12] Y. N. V. Reddy , K. E. Koepp , R. Carter , et al., “Rate‐Adaptive Atrial Pacing for Heart Failure With Preserved Ejection Fraction: The RAPID‐HF Randomized Clinical Trial,” Journal of the American Medical Association 329, no. 10 (2023): 801–809, 10.1001/jama.2023.0675. PMC9986839 36871285

[13] M. J. Page , J. E. McKenzie , P. M. Bossuyt , et al., “The PRISMA 2020 Statement: An Updated Guideline for Reporting Systematic Reviews,” British Medical Journal 372 (2021): n71, 10.1136/bmj.n71. PMC8005924 33782057

[14] X. Wan , W. Wang , J. Liu , and T. Tong , “Estimating the Sample Mean and Standard Deviation From the Sample Size, Median, Range and/or Interquartile Range,” BMC Medical Research Methodology 14 (2014): 135, 10.1186/1471-2288-14-135. PMC4383202 25524443

[15] J. A. C. Sterne , J. Savovic , M. J. Page , et al., “RoB 2: A Revised Tool for Assessing Risk of Bias in Randomised Trials,” British Medical Journal 366 (2019): l4898, 10.1136/bmj.l4898. 31462531

[16] G. A. Wells , B. Shea , D. O'Connell , et al., “The Newcastle‐Ottawa Scale (NOS) for Assessing the Quality of Nonrandomised Studies in Meta‐Analyses,” (2011), Ottawa: Ottawa Hospital Research Institute.

[17] M. Infeld , K. Wahlberg , J. Cicero , et al., “Effects of Continuous Accelerated Pacing on Cardiac Structure and Function in Patients With Heart Failure With Preserved Ejection Fraction: Insights From the myPACE Randomized Clinical Trial,” Journal of the American Heart Association 13, no. 9 (2024): e032873, 10.1161/JAHA.123.032873. PMC10863817 38156545

[18] M. Subramanian , S. Yalagudri , D. K. Saggu , et al., “Accelerated Bachmann Bundle Area Pacing for Atrial Resynchronization in Patients With Non‐Obstructive Hypertrophic Cardiomyopathy and Heart Failure With Preserved Ejection Fraction: A Randomized Crossover Trial,” Heart Rhythm 22 (2025): 2757–2765, 10.1016/j.hrthm.2025.07.028. 40714334

[19] N. Habel , K. Wahlberg , M. Infeld , et al., “Physiologic Accelerated Pacing as a Treatment for Heart Failure With Preserved Ejection Fraction (Pace‐Hfpef): A Prospective, Single‐Center, Randomized Multiple‐Crossover Study [Abstract],” (2024).

[20] K. Wahlberg , M. Arnold , D. Lustgarten , and M. Meyer , “Higher Resting Heart Rate Improves Heart Failure Symptoms and Exercise Capacity in Patients With Diastolic Dysfunction [Abstract],” Journal of the American College of Cardiology 73, no. 9 Suppl 1 (2019): 745, 10.1016/S0735-1097(19)31352-4.

[21] T. A. McDonagh , M. Metra , M. Adamo , et al., “2021 ESC Guidelines for the Diagnosis and Treatment of Acute and Chronic Heart Failure,” European Heart Journal 42, no. 36 (2021): 3599–3726, 10.1093/eurheartj/ehab368. 34447992

[22] J. F. Nauta , Y. M. Hummel , P. van der Meer , C. S. P. Lam , A. A. Voors , and J. P. van Melle , “Correlation With Invasive Left Ventricular Filling Pressures and Prognostic Relevance of the Echocardiographic Diastolic Parameters Used in the 2016 ESC Heart Failure Guidelines and in the Diagnostic Algorithm for Heart Failure With Preserved Ejection Fraction,” European Journal of Heart Failure 20, no. 9 (2018): 1303–1311, 10.1002/ejhf.1220. 29877602

[23] E. Wolsk , D. Kaye , B. A. Borlaug , et al., “Resting and Exercise Haemodynamics in Relation to the Six‐Minute Walk Test in Patients With Heart Failure and Preserved Ejection Fraction,” European Journal of Heart Failure 20, no. 4 (2018): 715–722, 10.1002/ejhf.976. PMC8000591 28949052

[24] T. van Loon , J. Rijks , J. van Koll , et al., “Accelerated Atrial Pacing Reduces Left‐Heart Filling Pressure: A Combined Clinical‐Computational Study,” European Heart Journal 45, no. 46 (2024): 4661–4674, 10.1093/eurheartj/ehae718. PMC11631061 39589540

[25] R. Zakeri , A. M. Chamberlain , V. L. Roger , and M. M. Redfield , “Temporal Relationship and Prognostic Significance of Atrial Fibrillation in Heart Failure Patients With Preserved Ejection Fraction,” Circulation 128, no. 10 (2013): 1085–1093, 10.1161/CIRCULATIONAHA.113.001475. PMC3910441 23908348

[26] J. A. Chirinos , P. Segers , T. Hughes , and R. Townsend , “Large‐Artery Stiffness in Health and Disease: JACC State‐of‐the‐Art Review,” Journal of the American College of Cardiology 74, no. 9 (2019): 1237–1263, 10.1016/j.jacc.2019.07.012. PMC6719727 31466622

[27] J. G. F. Cleland , K. V. Bunting , M. D. Flather , et al., “Beta‐Blockers for Heart Failure With Reduced, Mid‐Range, and Preserved Ejection Fraction: An Individual Patient‐Level Analysis of Double‐Blind Randomized Trials,” European Heart Journal 39, no. 1 (2018): 26–35, 10.1093/eurheartj/ehx564. PMC5837435 29040525

[28] D. N. Silverman , T. B. Plante , M. Infeld , et al., “Association of Beta‐Blocker Use With Heart Failure Hospitalizations and Cardiovascular Disease Mortality Among Patients With Heart Failure With Preserved Ejection Fraction,” JAMA Network Open 2, no. 12 (2019): e1916598, 10.1001/jamanetworkopen.2019.16598. PMC6902757 31800067

[29] M. Infeld , “Accelerated Physiologic Pacing in Patients With Heart Failure With Preserved Ejection Fraction: An Argument in Support of Therapeutic Heart Rate Modulation [Perspective],” Heart Rhythm O2 5, no. 4 (2024): 327–333, 10.1016/j.hroo.2024.03.009. PMC11148487 38840759

[30] M. Meyer , “Invited Commentary: Heart Rate as a Therapeutic Target in Heart Failure With Preserved Ejection Fraction [Editorial],” European Heart Journal 45, no. 46 (2024): 4965–4967, 10.1093/eurheartj/ehae766. 39589479

[31] S. Dhont and P. B. Bertrand , “Heart Rate Modulation in Heart Failure With Preserved Ejection Fraction: Lessons From Nature,” European Journal of Heart Failure 28, no. 2 (2026): 169–171, 10.1002/ejhf.3016. 41771099

[32] A. Ababei , L. A. Hrib , A. C. Iancu , A. Khebbaiz , R. Vatasescu , and S. Bogdan , “Anti‐Bradycardia Pacing‐Impact on Patients With HFpEF: A Systematic Review,” Heart Failure Reviews 29, no. 2 (2024): 523–534, 10.1007/s10741-024-10382-1. PMC10942895 38282011

