# Supplementary material for: Hemodynamic and Functional Outcomes of Rate‐Adaptive and Physiologic Pacing in Heart Failure With Preserved Ejection Fraction: A Systematic Review and Meta‐Analysis
Source: J Arrhythm. 2026 Oct 23;42(5):e70504. doi: 10.1002/joa3.70504 (PMC13694742; doi:10.1002/joa3.70504)
Supplement: Supplementary file 1 [file JOA3-42-e70504-s001.zip › FigS1.docx]

**Supplementary Material**

**Supplementary Figures S1-S11.** Funnel plots for each pooled outcome, presented for visual inspection only (formal small-study/publication-bias testing was not performed because no outcome included >=10 studies).
